# Expression of undifferentiated embryonic cell transcription factor-1 (UTF1) in breast cancers and their matched normal tissues

**DOI:** 10.1186/s12935-014-0116-6

**Published:** 2014-11-12

**Authors:** Chaoyang Xu, Ying Zhou, Wei Chen

**Affiliations:** Department of Breast and Thyroid Surgery, Shaoxing People’s Hospital, Shaoxing Hospital of Zhejiang University, Shaoxing, Zhejiang 312000 China; Department of Medical Research Center, Shaoxing People’s Hospital, Shaoxing Hospital of Zhejiang University, Shaoxing, Zhejiang China

**Keywords:** Breast cancer, UTF1, Polymerase chain reaction (PCR), Metastasis, Lymph node

## Abstract

**Objectives:**

Undifferentiated embryonic cell transcription factor-1 (UTF1) plays a critical role in the developmental timing during embryonic development. However, there is little paper dealing with UTF1 expressed in adult tissues. In the present study, we evaluate the expression of UTF1 in breast cancer and its correlation with clinicopathological parameters.

**Methods:**

Real-time polymerase chain reaction (real-time PCR) was applied to detect the expression of UTF1 mRNA in the 55 pairs of samples of breast cancer tissues and match normal tissues. △△CT method was used to evaluate the relative quantity of target mRNA expression.

**Results:**

Among the 55 pairs of samples of breast cancer tissues and match normal tissues adjacent to the tumor, the UTF1 mRNA levels in normal tissues were significantly higher than those observed in breast cancer tissues (p < 0.001). UTF1 mRNA levels expression correlated with lymph node metastasis (p = 0.002) and tumor size (p < 0.001).

**Conclusions:**

Expression of UTF1 in breast cancer tissues were confirmed in this study. Decreased expression of UTF1 mRNA in breast cancer tissues was maybe one of the factors impact on tumorigenes in breast cancer patients.

## Introduction

Currently breast cancer remains one of the most common cancers worldwide, and one of the leading causes of cancer related death in the word. Identification of biological markers ability to predict risk of prognosis in breast cancer is limited. To improve the clinical outcome of patients with advanced breast carcinoma, it is necessary to target novel biomarker genes, which appear to be involved in carcinoma development, as we had described previously [1,2].

Previous reports have indicated that there was a possible correlation of undifferentiated embryonic cell transcription factor-1 (UTF1) with the biological of cancer cell [3]. And the low expression of UTF1 may connect with poorer prognosis among patients with cervical cancer [4].

Undifferentiated embryonic cell transcription factor-1 (UTF1), a transcriptional co-activator, was first isolated and identified from mouse F9 embryonic carcinoma cells [5]. The human UTF1 gene is located on chromosome 10q26 and consists of two exons and one short intron, which plays a critical role in the developmental timing during embryonic development [6,7]. UTF1 is readily expressed in embryos and embryonic stem cells, but it is either undetectable or its expression remains at low levels in normal adult tissues, suggesting that UTF1 may play a critical role in cell proliferation and/or differentiation during embryonic development.

Recently, UTF1 confirmed the ability to facilitate the reprogramming of human somatic cells to induced pluripotent stem (iPS) cells [8]. Thus, UTF1 plays an important role in stem cells, and the expression of UTF1 cancer cells may have stem cell-like features. This prompted us to evaluate UTF1 status in human beast cancer. In addition, UTF1 has been reported to bind to a specific subset of mRNAs and modulate many gene translations, including those for p27 and Oct-3/4 [9].

The expression of UTF1 was subsequently detected in germ cell neoplasms [10], and cervical cancer [4]. Although UTF1 has been extensive studied in other cancers, no investigation has been conducted in breast cancer. We hypothesize that UTF1 may play a role in the oncogenesis of breast cancer. The present study was undertaken to examine the expression of UTF1 in breast cancer tissues by using a validated specific and sensitive real-time quantitative PCR assay. Here, we examined fifty-five cases of breast cancer tissues for the potential difference of UTF1 expression in breast cancer tissues and their matched normal tissues, and to assess the relationship between UTF1 expression and clinicopathological parameters in breast cancer patients.

## Materials and methods

### Case selection

Specimens were obtained from 55 patients who underwent surgery of breast cancer at the Department of Breast and Thyroid Surgery of Shaoxing Hospital of Zhejiang University, between July 2012 and November 2013. Informed consent was obtained from all patients, and the study was conducted according to the guidelines of the Hospital Ethics Committee. The patients aged 35 to 73 years (mean 58.7 years). The correlation between the expression of UTF1 and clinicopathologic parameters including age, differentiation statue and pTNM pathological classification according to the International Union against Cancer (UICC) were evaluated. The clinicopathologic feature of the 55 cases were summarized in Table 1

**Table 1:** **Clinicopathologic characteristics of 55 cases of patients with breast cancer**

**Clinicopathologic parameter**	**Case (n)**	**%**
Age		
≦ 35	5	9.1%
> 35	50	90.9%
Tumor size (cm)		
≦ 2	36	65.5%
> 2	19	34.5%
Histological grading		
I-II	9	16.4%
III	46	83.6%
Hormone-receptor		
ER (+)/PR (+)	23	41.8%
ER (+)/PR (−)	7	12.7%
ER (−)/PR (+)	4	7.3%
ER (−)/PR (−)	21	31.2%
Lymph node metastasis		
Yes	24	43.6%
No	31	56.4%

.

### RNA extraction and cDNA synthesis

Total RNA was extracted from fleshly frozen gastric tissues using the Trizol reagent (Invitrogen life Technologies, USA). Total RNA was reverse reverse-transcribed into single-strand complementary DNA (cDNA) using moloney-murine leukemia (M-MLV) reverse transcriptase (Promega, Madison,WI, USA). Briefly, the RNAwas denatured by heating for 5 min at 70°C, cooled on ice, and then used for reverse transcription (2 μg of total RNA, 25U of RNAse inhibitor, 0.5 mM each of dNTPs, 1.5 μM reverse primer and 200U of M-MLV reverse transcriptase in a total volume of 25 μl). For reverse transcription, tubes were incubated at 42°C for 60 min, followed by rapid cooling.

### Real-time quantitative PCR

Real-time RT-PCR analyses were performed with the ABI Prism 7500 sequence detection system (Applied Biosystems, Foster City, CA). 25 μl reaction mixture containing 2 μl of cDNA template, 1 μl each of sense and anti-sense primers, 1 × SYBR Green Universal PCR Mix was amplified as follow: denaturation at 95°C for 10 min and 40 cycles at 95°C for 30 s, 55°C for 30 s and 72°C for 40 s. Real-time quantitative PCR reaction was performed in triplicate for each sample and a mean value of glyceraldehyde 3-phosphate dehydrogenase (GAPDH) used to calculate mRNA levels. Quantitative analysis was performed using the comparative CT method [11,12]. The UTF-1 mRNA copy numbers in normal and tumor tissues were normalized to mRNA copy numbers of the house keeping gene, GAPDH to give a value △CT. This final value was to determine changes in expression of UTF-1 in each sample. The primer sequences for UTF-1 were as follows: forward primer 5'- ATGGGGCTGCTGGGCGACAACG-3', reverse primer 5'- GGGGAGGCGTCCGCAGACTTCG-3'. The primers for GAPDH were taken from a previously published assay [13]. Fluorescent data were converted into RQ measurements, which stand for relative expression automatically by the SDS system software and exported to Microsoft Excel. Thermal dissociation plots were examined for biphasic melting curves, indicative of whether primerdimers or other nonspecific products could be contributing to the amplification signal.

### Statistical analysis

Statistical analysis was conducted using the statistical program SPSS 15.0 for Windows (SPSS, Chicago, IL, USA). Pre-treatment characteristics were analyzed using the 2-tailed χ2 test. The 2-tailed t-test was used to evaluate the correlation between UTF-1 expression and clinicopathologic parameters.

## Results

### Expressions of UTF-1 mRNA in breast cancer tissues by real-time PCR

As shown in Table 2, of the 55 samples of breast cancer tissues and matched normal tissues adjacent to the tumor, expression of UTF-1 was detected in total samples. In normal tissues, the UTF-1 mRNA levels ranged from 0.039 to 41.96 with a median of 20.16. In breast cancer tissues, the UTF-1 mRNA levels ranged from 0.008 to 6.263 with a median of 1.517. The UTF-1 mRNA levels in normal tissues were significantly higher than those observed in breast cancer tissues (p < 0.001).

**Table 2:** **Relative quantity of UTF-1 mRNA in breast cancer tissues and match normal tissues**

	**Tumor tissue**	**Normal tissues**	**P**
Relative UTF-1 expression	1.97 ± 0.161	7.47 ± 1.01	<0.001

### Correlation between UTF-1 and clinicopathological parameter

According to median expression level of UTF-1, 55 cases of breast cancer patients were divided into two groups, high UTF-1 expression group (UTF-1 expression level >1.517) and low UTF-1 expression group (UTF-1 expression level ≦ 1.517). The mean number of metastasis lymph nodes in high UTF-1 group is lower than low UTF-1 expression group (0.33 vs. 1.75), the differences among them had statistically significant (p = 0.002) (Table 3). Moreover, tumor size was different between high expression UTF-1 versus low expression UTF-1 (1.34 vs. 2.46), the difference also have statistically significant (p < 0.001) (Table 3).

**Table 3:** **Correlation between the expression of UTF-1 mRNA and number of metastasis lymph nodes, tumor size**

	**UTF-1 expression level ≦1.517**	**UTF-1 expression level >1.517**	**P**
Number of metastasis lymph nodes	0.33 ± 0.56	1.75 ± 2.12	p = 0.002
Tumor size	1.34 ± 0.60	2.46 ± 1.28	(p <0.001)

## Discussion

In the present study, we found the relative quantity of UTF-1 mRNA mRNA in 55 samples of breast cancer tissues as determined by real-time PCR. UTF-1 mRNA was expressed more predominantly in normal tissues adjacent to the tumor than it was expressed in breast cancer tissues. We showed that UTF-1 was lower expressed in breast cancer tissues than corresponding normal tissues. Previous studies have reported that UTF-1 is expressed in various human epithelial-type neoplasias, such as cervical cancer [4]. At present, Mouallif et al. evaluated UTF1 expression levels immunohistochemically in eight normal epithelia including breast (prostate, endometrium, bladder, colon, oesophagous, lung and kidney) and their corresponding tumors in a recent paper [14].

In the current study, real-time quantitative PCR was used to compare the expressions of UTF-1 mRNAs in 55 breast cancers and their matched normal tissues, which allows detecting UTF-1expression in breast tissues with very low level. Furthermore, we correlated these findings with the clinicopathological parameter of the breast cancers. The result showed that the low expression level of UTF-1 mRNAs in breast cancer patients was significantly associated with metastasis lymph nodes and tumor size. Our present study clarify that UTF-1 protein could act as a potential biomarker for prognosis assessment of breast cancer. Related mechanism is worthy of further investigation.

Previous studies have shown the expression of UTF-1 cells was identified in a subpopulation of stem cell-like cells [15] and the expression of UTF-1 may influence the susceptibility of cancer cells to chemotherapeutic agents and radiotherapy. These data suggested that positive expression of UTF-1 impact on cancer patients’ survival not only influence the susceptibility of cancer cells to chemotherapeutic agents, but also other mechanism such as cell proliferation [16], stem cell-like features [17,18].

Recently, it was reported that the UTF1-overexpressing cells transactived p27Kip1 resulting in G1/S arrest, which may provide an explanation for UTF1 functions in cervical carcinogenesis. The similar results may present in breast cancer patients. In summary, our study demonstrated that the expression level of UTF1 may play a role in the oncogenesis and may serve as a biomarker for prognosis. Based on our findings, UTF1 gene could be a potential target for the treatment of breast cancer. The findings of this study present a novel knowledge of UTF1 and a potential future prospect for breast cancer treatment.
